# Pedestrian Road Traffic Injuries in Urban Peruvian Children and Adolescents: Case Control Analyses of Personal and Environmental Risk Factors

**DOI:** 10.1371/journal.pone.0003166

**Published:** 2008-09-10

**Authors:** Joseph Donroe, Monica Tincopa, Robert H. Gilman, Doug Brugge, David A. J. Moore

**Affiliations:** 1 Fogarty International Center/ Ellison Medical Foundation Research Fellow, Asociación Benéfica PRISMA, San Miguel, Lima, Perú; 2 Johns Hopkins School of Medicine, Baltimore, Maryland, United States of America; 3 Department of International Health, Johns Hopkins Bloomberg School of Public Health, Baltimore, Maryland, United States of America; 4 Asociación Benéfica PRISMA, San Miguel, Lima, Perú; 5 Laboratorio de Investigación de Enfermedades Infecciosas, Universidad Peruana Cayetano Heredia, San Martín de Porras, Lima, Perú; 6 Tufts University School of Medicine, Boston, Massachusetts, United States of America; 7 Department of Infectious Diseases and Immunity, Imperial College London Wellcome Centre for Clinical Tropical Medicine, Faculty of Medicine (Hammersmith Campus), London, United Kingdom; 8 Laboratorio de Investigación de Enfermedades Infecciosas, Universidad Peruana Cayetano Heredia, San Martín de Porras, Lima, Perú; 9 Asociación Benéfica PRISMA, San Miguel, Lima, Perú; University of Bern, Switzerland

## Abstract

**Background:**

Child pedestrian road traffic injuries (RTIs) are an important cause of death and disability in poorer nations, however RTI prevention strategies in those countries largely draw upon studies conducted in wealthier countries. This research investigated personal and environmental risk factors for child pedestrian RTIs relevant to an urban, developing world setting.

**Methods:**

This is a case control study of personal and environmental risk factors for child pedestrian RTIs in San Juan de Miraflores, Lima, Perú. The analysis of personal risk factors included 100 cases of serious pedestrian RTIs and 200 age and gender matched controls. Demographic, socioeconomic, and injury data were collected. The environmental risk factor study evaluated vehicle and pedestrian movement and infrastructure at the sites in which 40 of the above case RTIs occurred and 80 control sites.

**Findings:**

After adjustment, factors associated with increased risk of child pedestrian RTIs included high vehicle volume (OR 7·88, 95%CI 1·97–31·52), absent lane demarcations (OR 6·59, 95% CI 1·65–26·26), high vehicle speed (OR 5·35, 95%CI 1·55–18·54), high street vendor density (OR 1·25, 95%CI 1·01–1·55), and more children living in the home (OR 1·25, 95%CI 1·00–1·56). Protective factors included more hours/day spent in school (OR 0·52, 95%CI 0·33–0·82) and years of family residence in the same home (OR 0·97, 95%CI 0·95–0·99).

**Conclusion:**

Reducing traffic volumes and speeds, limiting the number of street vendors on a given stretch of road, and improving lane demarcation should be evaluated as components of child pedestrian RTI interventions in poorer countries.

## Introduction

Road traffic injuries (RTIs) are an important cause of morbidity and mortality, and are projected to become the sixth leading cause of death and third leading cause of disability adjusted life years (DALYs) lost globally by the year 2020 [1]. Poorer nations are disproportionately affected by RTIs and account for approximately 85% of RTI deaths and 90% of RTI disability [2]. In poorer countries of Latin America, RTIs are already the sixth leading cause of death and third leading cause of morbidity for all ages [3]. While well-designed research, successful interventions, and legislative priority has led to a substantial decrease in the burden of RTIs in wealthier regions, the rates of RTIs in many poorer nations are increasing [4].

Children and pedestrians are especially vulnerable to traffic injuries, particularly in developing countries [5], [6], [7], [8], [9]. In the low to middle income countries of the Americas, RTIs are the number one cause of death and morbidity for children aged 5–14, and a leading cause of death for children aged 0–4 [3]. Additionally, the RTI fatality rate for children of poorer countries is as much as six times that of children from high income countries [5]. Pedestrians are involved most frequently in RTIs in the developing world, and represent up to 54% of those injured in Latin American studies [8], [10], [11].

Prevention of child pedestrian RTIs has focused on modifying both personal (education initiatives) and, more effectively, environmental (traffic calming) risk factors [12], [13], [14], [15], [16], [17], [18]. Environmental risk factors themselves, however, have been less rigorously studied [19]. The personal risk factors encountered in the literature include age, gender, household overcrowding, poverty, single parent homes, and low levels of education in caregivers, while environmental risk factors include high traffic volumes, high vehicle speeds, presence of sidewalks, and density of curb side parking [12], [15], [16], [17], [19], [20], [21], [22], [23], [24], [25]. The overwhelming majority of these studies were conducted in developed countries [4], [26], and the results are commonly relied upon when importing or creating intervention strategies for the developing world. The assumption, however, that developed world practices translate into effective prevention measures in poorer countries may be erroneous as they may not be affordable, may require disproportionate technologies, and may miss important risk factors unique to developing world settings [14], [27], [28].

The aim of this study was to assess personal and environmental risk factors for child pedestrian RTIs in the urban, developing world setting of Lima, Peru. Our intention is to aid the design of new RTI interventions or the translation of existing ones from high income nations to poorer ones based on locally relevant risk factors.

## Materials and Methods

### Study Design and Setting

This analysis is a sub-study of a large, community based cross sectional study of childhood injuries in San Juan de Miraflores (SJM), a poor, urban district of Lima, Peru. It includes results from the cross sectional study and two nested case control studies exploring personal and environmental risk factors for child pedestrian RTIs. Studies were conducted between January 2005 and July 2006.

### Participants

#### Cross sectional and personal risk factor case control studies

In the cross sectional study, six health promoters with high school graduate level education administered door to door surveys in 12 SJM zones, divided along existing neighbourhood borders. Staff began randomly and proceeded until each zone was completed. Households with a consenting adult and at least one resident child aged ≤18 were eligible.

In the personal risk factor case control study, health promoters administered follow up surveys to cases of child pedestrian RTIs and randomly selected age and gender matched controls from the original study. Subjects were recruited to a goal of 100 cases and 200 controls. Cases were children who incurred a RTI during pedestrian activity in SJM from the year 2000 onward. RTIs occurring in parking lots, driveways, or while the vehicle was reversing were excluded. Two controls per case were selected by random assortment of all potential controls, and then random number generation to identify the first and subsequent controls. Controls were included if they could be age (within one year) and gender matched to a case, and there was no family history of pedestrian RTI. There was no compensation for participation.

#### Environmental case control study

The environmental case control study used the same case-control sets as in the personal risk factor study. Goal recruitment was 40 case and 80 control environments. Case sites of RTIs were included if the environment in which the RTI occurred had not changed since the time of the injury (as reported by the guardian of the injured child), and the RTI occurred between the hours of 6:00 and 20:00 (to minimize personal safety risk to staff as the district in which the study took place was quite dangerous). Control environments were selected by considering the origin and destination of the case child prior to injury, and the distance (number of blocks) from the origin that the RTI occurred. As an example, if a case was injured travelling from his local market to home, three blocks from the market, we then identified which market the control subject normally visited, and the route they would take from the market to home. The control site was then three blocks from their market, on their route home.

### Data Collection

All study surveys were extensively pilot tested, and completed surveys were reviewed by the principal investigator (JD).

#### Cross sectional and personal risk factor case control studies

In the cross sectional study we administered a semi-structured survey including the core data sets recommended by the WHO [29]. Children aged ≥12 years and present at the time of the survey were interviewed in the presence of a guardian. If the child was not present or was <12 years of age, a guardian was interviewed. The personal risk factor study required that the injured child and guardian be present at the time of interview and collected demographic data and school information in relation to the year in which the injury (or case injury for controls) took place and precise RTI time and location data for cases.

#### Environmental case control study

In the environmental case control study, structured, 1.5 hour assessments were performed. Briefly, we evaluated: pedestrian movement, including volume, use of cross walks, and street vendor density; vehicle movement, including speed, vehicle specific volumes (cars, trucks, public transportation, motorcycle taxis), and traffic code infractions; pedestrian infrastructure, including sidewalks, crosswalks, and crossing lights; vehicle infrastructure, including road conditions, traffic lights, speed bumps, lane demarcation, and curb side parking.

Environmental assessments were performed on the same day of the week and at the same time of day as when the case injury occurred. Case and control assessments were done simultaneously by two health promoters per site. Measurements were made only in the direction the vehicle was travelling prior to causing the case injury, on a section of road extending 150 meters from the injury site. All measurements were made by direct observation. Vehicle speed was recorded during a dedicated 30 minute period using digital timers to measure the time to traverse 150 meters.

### Definitions

We defined *pedestrian activity* as walking, running, or standing, but not cycling or skating. A *road traffic injury (RTI)* was any unintentional injury inflicted by a motorized vehicle. A *serious RTI* necessitated a healthcare consultation, i.e. visit to a hospital or health post. Of the RTIs identified in the cross sectional study, only serious RTIs were included in the case control studies. We considered *poverty* to be *present* if one or more of the following criteria were fulfilled: economic dependence (defined in Peru as ≥3 household occupants per wage earner), absence of indoor plumbing, dirt floors, and children in the home aged 6–12 not attending school. We considered *overcrowding* to be ≥4 people per room, excluding the kitchen, bathroom, and hallways. *Education in the head of household* and *maternal education* were *low* if primary schooling was incomplete. *Years of residence* refers to the total number of years in which the family has lived in the home where the interview took place. *Speeds and volumes* (i.e. pedestrian, vehicle) were considered *high* if they were in the highest tertile of recorded measurements. *Avenues*, *streets*, and *roads*, were defined according to city planning maps. *Street vendors* were sidewalk or street merchants without fixed locals.

### Statistics

Sample size in the personal risk factor study was calculated with an assumed exposure in the control population of 50%, lowest detectable OR of 2.15, alpha equal to 0.05 and a power of 80 percent. Sample size in the environmental risk factor study was limited by time and funding considerations. Statistical analysis was performed using SPSS software (SPSS ®, SPSS Inc, ver. 11.5, Chicago, Illinois) and StatsDirect statistical software (StatsDirect Ltd ®, ver. 2.7.0, UK). Descriptive analyses determined the proportion and percentages of occurrences for binary and categorical variables. Conditional logistic regression methods were used to generate unadjusted matched odds ratios (OR) with 95% confidence intervals (CI) for each exposure variable and adjusted multivariate models for both personal and environmental risk factors. The best fitting multivariate models were built beginning with two variables with historic association to the outcome based on the review of the literature (poverty and maternal education for the personal risk factor study, and vehicle speed and volume for the environmental risk factor study), then adding subsequent variables if they improved the model. Improved models were those in which the −2 log likelihood ratio was greater than a critical value derived from the chi-square distribution table based on degrees of freedom at an alpha level of 0.05. Variables evaluated in the multivariate analysis but eliminated by statistical criteria are listed in Appendix S1. All statistical tests were two-sided and p-values <0.05 were considered statistically significant. Interactions between selected factors were tested however none were found to be significant.

### Ethics

The study protocols, informed consent and assent forms, and data collection instruments were approved by the human research ethics committee of Asociación Benéfica PRISMA (FWA 00001219). All interviewed children signed written assent forms and one guardian per household provided written consent for the cross sectional and case control portions of the study.

## Results

### Descriptive Analysis

**Figure 1:**
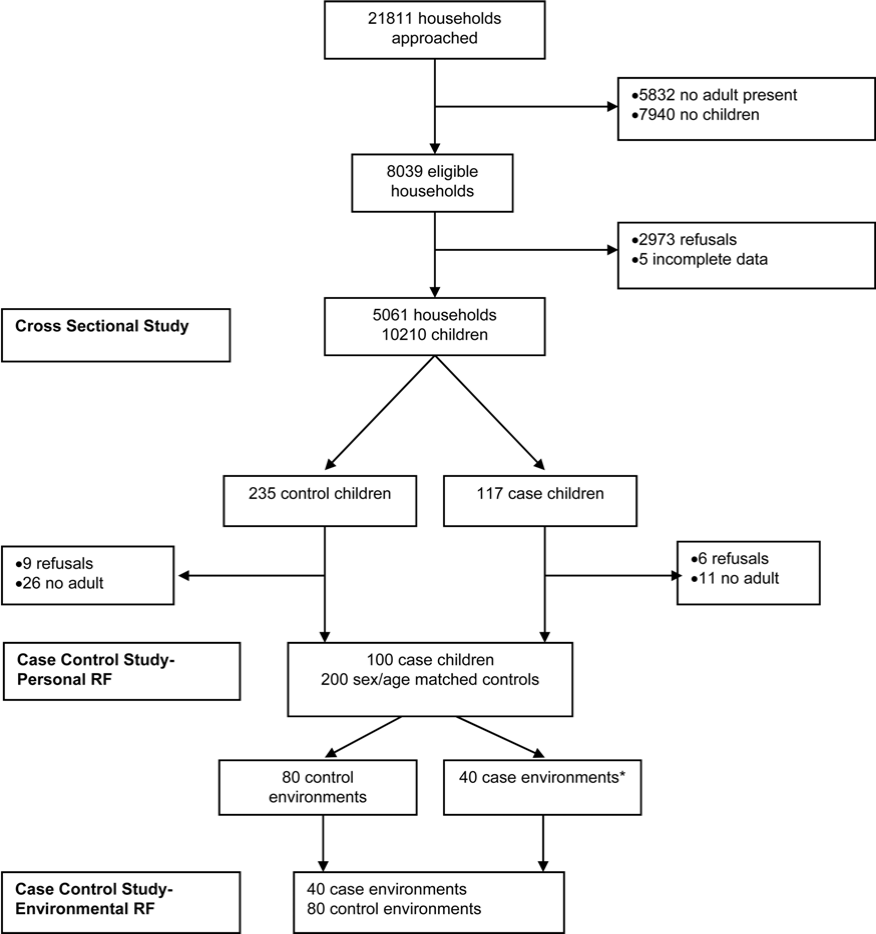
In the cross sectional study, 24% (5832) and 36% (7940) of homes approached were not surveyed because no adult was home after two attempts or there were no resident children, respectively. Of the remaining 8039 households, 2973 (37%) refused to participate and five households (12 children) were excluded due to incomplete data. In the personal risk factor case control study, health promoters made two visits to homes in which no one was present, after which time the case or control was replaced. In 37 instances no adult was present on the repeat visit. Of the 40 cases selected for the environmental analysis, 93% were injured within the two years preceding the study. *Traffic volumes were too high to allow for accurate measurement in 2 case environments, thus they and their corresponding control environments were removed and replaced. RF = Risk Factors.

Participant recruitment and attrition are highlighted in Figure 1. In the cross sectional study, 21811 households were approached, of which 8039 were eligible for participation. Of these, 63% consented and were surveyed and the final analysis included 5061 households and 10210 children (median two per household; characteristics described in Table 1). Between 2000 and 2005, this population sustained 141 pedestrian RTIs, of which 117 (83%) were serious. No surveyed household reported child pedestrian RTI deaths during this period. In the personal risk factor study, there were four instances in which cases were clustered within households (two cases per household). Of these four homes, 50% (2·0) were poor, none had a low level of maternal education, there was a median of four resident children (range 2·0 to 5·0), and the median time of family residence was 11·5 years (range 2·0 to 15·0).

**Table 1:** Cross sectional study population characteristics (n = 10210).

Characteristic	N	Percent
Gender-male	5269	51·6
Poverty- present	5635	55·2
Education of head of household-low*	1951	19·1
Overcrowding-yes	785	7·7
Ages (years)†		
<1	461	4·5
1–4	2297	22·5
5–9	2864	28·1
10–14	2790	27·3
15–18	1797	17·6

* 9 missing values.

† 1 missing value.

Characteristics of serious pedestrian injuries are presented in Table 2. In the 1–4 and 5–9 year old groups, RTIs most commonly occurred during trips to or from the market (50% and 40%, respectively) and playing in the street (27% and 28%, respectively). In the 10–14 year old group, RTIs most commonly involved trips to or from school (28%), the market (23%) or a relative's home (23%). Younger children (aged 1–9) were more commonly injured while crossing in non designated areas (70% of RTIs in this age group).

**Table 2:** RTI Characteristics (Cross Sectional Study).

Characteristics of serious RTI	N	Percent
RTI requiring hospital/clinic visit*	117	
<12 hours	86	76·1
>12 hours	27	23·9
Median days of hospitalization (n = 27)	3	
**Age at time of serious RTI (years)**		
<1	0	-
1–4	22	18·8
5–9	47	40·2
10–14	39	33·3
15–18	9	7·7
**Activity**		
Median distance from home (blocks)	2·5	
Median distance from point of origin (blocks)	0·5	
RTI occurred during:		
Trip to/from store/market	41	35·0
Trip to/from school	18	15·4
Trip to/from relative's home	14	12·0
Play	24	20·5
Other†	20	17·1
**Alone or with another minor**	82	70·1
**Time of day**		
Morning	33	28·2
Afternoon or evening	84	71·8
**Location**		
Road or street	46	39·3
Avenue	70	59·8
Highway	1	0·9
**Agent**		
Taxi or mototaxi	48	41·0
Private auto	27	23·1
Public transportation (bus, minibus, or van)	26	22·2
Other (truck, motorcycle)	16	13·7

* 4 missing values for duration of hospital visit; percentages shown are of the 113 with known duration.

† Includes to/from friends home, bus stop, restaurant, parent's work, park, movies, and unspecified purpose.

Of the 120 evaluated sites, only one had a traffic light, two had stop signs, and there were no posted speed limits. Tertiles derived from aggregate case and control data for pedestrian volume and vehicle speed and volume were used to define strata in the risk factor analyses. The middle pedestrian volume tertile was 101 to 201 pedestrians/hr, the middle vehicle speed tertile was 33.9 to 44.6 km/hr, and the middle vehicle volume tertile was 24 to 249 vehicles/hr. The median number of vehicles per hour at the case sites was 244·0 (range 0·0 to 4503·0), composed of motorcycle taxis (29%), public transportation (buses and vans- 28%), taxis (26%), and cars (15%). At control sites, the median number of vehicles per hour was 66·0 (range 0·0 to 1212·0), with a similar profile composed of motorcycle taxi (33%), public transportation (25%), taxi (25%), and cars (13%). The median vehicle velocity at case and control sites was 40·7 km/hr (range 18·2 to 60·5) and 39·8 km/hr (range 20·4 to 83·1), respectively.

### Risk Factor Analyses

Univariate associations between case and control characteristics and pedestrian RTI are presented in Table 3. More hours per day in school was protective over pedestrian RTI, while larger streets, commercial or mixed commercial zones, and high vehicle volumes were associated with increased odds of pedestrian injury.

**Table 3:** Characteristics of case and control participants and sites in the risk factor analyses*.

Characteristic	Cases (%)	Controls (%)	Matched OR (95% CI)
**Personal risk factor analysis**	100	200	
Gender- male	67 (67·0)	134 (67·0)	NA
Age- mean±SD	11·5±4·4	11·9±4·1	NA
Poverty- present	47 (47·0)	103 (51·5)	0·86 (0·52–1·41)
Maternal education- low	10 (10·0)	9 (4·5)	1·86 (0·73–4·75)
Overcrowding- yes	12 (12·0)	12 (6·0)	2·00 (0·90–4·45)
Number of resident children- median (range)	2·0 (1·0–9·0)	2·0 (1·0–8·0)	1·18 (0·99–1·42)
Years of family residence- median (range)	10·5 (0·1–46·0)	14 (0·2–50·0)	0·98 (0·96–1·00)
Hours/day in school- mean±SD†	**4·8±0·96**	**5·1±0·77**	**0·56 (0·37–0·83)**
Attend school mostly in the afternoon- yes†	27 (29·3)	42 (21·8)	1·37 (0·79–2·40)
Walks to school- yes†	83 (89·0)	164 (85·0)	1·36 (0·64–2·91)
Allowed to play in street- yes†	40 (40·0)	67 (33·7)	1·38 (0·82–2·32)
Family has car- yes	13 (13·0)	38 (19·0)	0·66 (0·35–1·27)
Home with yard- yes	66 (66·0)	128 (64·0)	1·09 (0·66–1·79)
Blocks from home to nearest park- median (range)†	1·0 (0·0–12·0)	1·0 (0·0–10·0)	0·98 (0·83–1·15)
**Environmental risk factor analysis**	40	80	
Time of day- morning	7 (17·5)	14 (17·5)	NA
Location- avenue	**28 (70·0)**	**35 (43·8)**	**3·90 (1·51–10·09)**
Zone- commercial or mixed commercial	**26 (65·0)**	**35 (43·8)**	**3·28 (1·24–8·65)**
Pedestrian volume- high‡	16 (40·0)	24 (30·0)	1·80 (0·79–4·09)
Vehicle volume- high‡	**20 (50·0)**	**20 (25·0)**	**6·49 (1·83–23·0)**
Vehicle Speed- high‡	18 (45·0)	22 (27·5)	2·10 (0·95–4·63)
Number of Street vendors- median (range)	0 (0–25)	0 (0–21)	1·21 (0·99–1·48)
Absent Lane Demarcation†	29 (74·4)	50 (62·5)	1·71 (0·73–4·02)
Dirt road- yes	3 (7·5)	13 (16·3)	0·44 (0·12–1·59)
Speed bump- yes	2 (5·0)	9 (11·3)	0·44 (0·10–2·06)
Gated community- yes	2 (5·0)	6 (7·5)	0·67 (0·13–3·30)
Pedestrian crosswalk present- yes	2 (5·0)	8 (10·0)	0·50 (0·11–2·35)
Sidewalk present- yes	27 (67·5)	62 (77·5)	0·63 (0·28–1·42)
>50% curbside parking	4 (10·3)	5 (6·3)	2·38 (0·38–14·97)
Park/play area nearby- yes	17 (42·5)	35 (43·8)	0·96 (0·47–1·94)

* Matched OR calculated with conditional logistic regression methods. OR denotes odd ratio, and CI confidence interval; NA- OR not calculated because cases/controls were matched by these variables.

† For hours/day in school and attends school mostly in pm, and walks to school-7 cases and 7 controls were not attending school at the time of injury; For allowed to play in street- 1 missing control value; For blocks from home to nearest park- 4 missing control values; For absent lane demarcation- 1 missing case value.

‡ High designation corresponds to the highest tertile of all values recorded at all 120 sites (vehicle volume≥250 vehicles/hr; vehicle speed≥44·7 km.hr, pedestrian volume≥201pedestrians/hr).

The final multivariate conditional logistic regression models for personal and environmental risk factors are shown in Table 4. More hours per day spent in school and years of family residency in the same home were protective against child pedestrian RTIs, while a greater number of children in the home, a greater number of street vendors, the absence of lane demarcations, and high vehicle volume and speed increased the odds of pedestrian injury.

**Table 4:** Multivariate associations between personal and environmental risk factors and pedestrian RTI*.

Risk Factor	Matched OR (95%CI)
**Personal**	
Poverty	0·58 (0·31–1·08)
Low Maternal Education Level	1·83 (0·65–5·17)
Number of resident children	**1·25 (1·00–1·56)** †
Years of residence	**0·97 (0·95–0·99)**
Hours/day in school‡	**0·52 (0·33–0·82)**
Attend school mostly in the afternoon‡	1·31 (0·70–2·46)
**Environmental**	
Vehicle volume- high§	**7·88 (1·97–31·52)**
Vehicle Speed- high§	**5·35 (1·55–18·54)**
Number of Street vendors	**1·25 (1·01–1·55)**
Absent Lane Demarcation∥	**6·59 (1·65–26·26)**

* OR denotes odd ratio, and CI confidence interval; Personal and environment al regressions were performed separately.

† p = 0·048.

‡ 7 cases and 7 controls were not attending school at the time of injury.

§ High designation corresponds to the highest tertile of all values recorded at all 120 sites (vehicle volume≥250 vehicles/hr; vehicle speed≥44·7 km.hr).

∥ 1 missing case value.

## Discussion

Effective intervention design, or translation of existing interventions, for child pedestrian injury prevention to the world's poorer countries requires identification of locally relevant modifiable risk factors. This study describes the context in which child pedestrian RTIs occurred in an urban district of a major Latin American city, and identified both personal and environmental risk factors. Briefly, and in order of increasing strength of association, we found that more years of family residence in the same location and longer length of the school day were protective, while a greater number of children living in the home, a greater number of street vendors, higher vehicle speeds, absent lane demarcation, and higher vehicle volumes increased the risk of child pedestrian RTI. Our study of personal risk factors is among few such case control studies in Latin America, and, to the best of our knowledge, the environmental case control component is the first reported from the developing world.

Personal risk factors for child pedestrian RTIs have been described extensively, but few are derived from case control studies in the developing world. We identified some similar associations, including familiarity with the local environment [20] (reflected by years of family residence) and the number of children living in the home [25]. However, other previously identified risk factors, such as poverty [15], [17], [20], [22], household crowding [12], [17], [20], [22], and low maternal education [20], [25] were not significant predictors within our population. SJM is a low income zone and the relative lack of economic variability may have weakened the effect of poverty. Overcrowding in this community may be compensated by the traditional Peruvian family dynamic in which multiple adult relatives live in the same or in neighbouring homes, increasing the number of caretakers per child. A similar effect may abrogate the anticipated importance of low maternal education. The protective effect of increased time spent in school has not been described previously. We found that each additional hour of school conferred a 48% decrease in the odds of a pedestrian RTI. This is unlikely a function of the added educational benefit of longer schooling, but rather a change in exposures. Children that are in school less may make more frequent trips to the market or spend more time playing in the street, the two most common exposures prior to RTI in this community. They may also be performing these activities at riskier times of day, when traffic patterns are more conducive to pedestrian RTI.

Environmental case control studies, designed to identify environmental risk factors amenable to modification, are rarely performed in pedestrian RTI investigations. One reason is the challenge inherent in selecting appropriate control sites. Prior studies have first chosen a control child and then the control site in relation to this child's home based upon the distance and/or direction that the case RTI occurred in relation to the case home [16], [21]. This method, however, fails to consider two important variables - the points of origin and destination of the case child prior to injury. Our innovative technique for control selection used distance as in previous studies, but with relation to the points of origin and destination of the case child prior to the injury. Our approach takes account of what children are doing when they are injured and tries to answer the question of why only one of the three children doing the same thing in different places should suffer an injury. This was a challenging undertaking and case selection was limited to those for whom the journey's start and end points could be clearly defined (e.g. home to school) so that adequate definition of the comparable control journey was feasible. We found strong associations between child pedestrian RTIs and higher traffic volume and higher vehicle speed, consistent with other studies from wealthier countries [15], [16], [21], [23]. Similar to studies by Roberts and Mueller, our final model incorporates vehicle speed as a categorical, rather than continuous, variable [15], [21]. The risk of injury to a child may be similar on a street where the average vehicle speed is 25 km/hr (lowest tertile) and 35 km/hr (middle tertile), while the risk increases once the average vehicle speed reaches 45 km/hr (highest tertile). The risk of injury, however, on a street where the average vehicle speed is 55 km/hr may not be significantly different from that of the street where the average speed is 45 km/hr, as both these values are above the critical speed limit. The increased risk of pedestrian RTI associated with a greater number of street vendors and absent lane demarcation has not previously been described. Street vendors may create hazardous conditions by obstructing portions of the street, diverting traffic, concealing oncoming vehicles from view, or distracting pedestrians and drivers. Absent lane demarcations likely contribute to disorderly traffic flow, perhaps leading to unpredictable traffic patterns and making it more difficult for children to judge safe times to cross the street. These two factors are particularly common in the developing world and may be important considerations when designing child RTI interventions there.

The strengths of this research include the large scale community based descriptive analysis and concurrent identification of both personal and environmental risk factors in the same community. The data are derived from and thus applicable to a developing world setting, addressing well recognized gaps in our understanding of child pedestrian RTIs in the world's highest risk regions. Finally, our novel approach to control environment selection makes an important contribution to the field of RTI research design. The low overall sampling rate of the cross sectional survey from which the cases and controls were generated should be considered when assessing the generalizability of the study. However, at the same time, the number of households and children surveyed was large. The difficult reality of conducting household surveys in a developing world setting is that childcare is often relegated to older siblings who cannot provide consent to participate and there exists a distrust of strangers grounded in the very tangible threat of home invasion and kidnappings. As only homes with at least one resident child were eligible for study inclusion, cases where an only child had been fatally injured in a RTI would have been missed, and, while likely a rare occurrence, this is a potential source of selection bias. Also, both case control studies involved injuries occurring up to five years prior to surveying, thus recall bias and changing roadside environments are important considerations. It is unlikely, however, that recall of socioeconomic and demographic factors would be biased significantly by having suffered an RTI. To minimize confounding by changing street environments, case families were asked if environmental modifications were made since the time injury. Affirmative or equivocal responses required selection of a new case site. Additionally, the sample size for the environmental risk factor study was small, thus increasing the probability of a type II error, and measurements were done by direct observation, therefore reported measures, while internally consistent and validated, should be considered approximations. Finally, children are only at risk for pedestrian RTIs when they are exposed to traffic while walking, therefore exposure factors (such time spent in school, walking to school, playing in the street, etc) are considered risk factors in our analysis. As the data is derived from case control studies, relationships between risk factors and outcomes represent associations and causality cannot be inferred.

Our findings have important public health implications. Most injuries affected children aged 5–9 years, and occurred while unaccompanied, going to or from market, playing in the street, and crossing in non designated areas suggesting room for targeted behavioural interventions as well as the need for local traffic calming measures. Taxis and motorcycle taxis were the most frequent vehicles responsible, thus law enforcement could have a particularly helpful role. In particular, motorcycle taxis are a common mode of transport in Lima, but drivers are often under-aged, vehicles are often filled beyond capacity, and they frequently violate traffic codes. The protective effect of time spent at school suggests the viability of longer school days or increased access to after school programs as a means of preventing child pedestrian injuries. Finally, our environmental analysis provides evidence to support measures to reduce traffic volumes and speeds, limit the number of street vendors on a given stretch of road, and improve lane demarcation.

It is clear that as disparities between high and low income countries continue to grow with regard to child pedestrian injuries, our goal should be to develop prevention strategies targeting risk factors relevant to a developing world context. This study advances that process by identifying both personal and environmental risk factors for child pedestrian RTIs in a major Latin America city.

## Supporting Information

Appendix S1 (0.02 MB DOC)
