# Supplementary material for: Pedestrian Road Traffic Injuries in Urban Peruvian Children and Adolescents: Case Control Analyses of Personal and Environmental Risk Factors
Source: PLoS One. 2008 Sep 10;3(9):e3166. doi: 10.1371/journal.pone.0003166 (PMC2528934; doi:10.1371/journal.pone.0003166)
Supplement: Appendix S1 — (0.02 MB DOC) [file pone.0003166.s001.doc]

**APPENDIX S1**

For the personal risk factor study, the following variables were eliminated from the final conditional regression model:

Overcrowding

Child works in the street

Child allowed to play in street

Total number of household occupants

Family ownership of a car

Distance to the nearest park from the child’s home

Presence of a yard

Number of days per week attending school

Method of travel to school (walk, transportation, both)

Number of streets crossed going/returning to/from school

Number of avenues crossed going/returning to/from school

Time spent walking to/from school

Accompanied by an adult (yes/no) while travelling to/from school

For the environmental study, the following variables were eliminated from the final conditional regression model:

Pedestrian volume

Volume of public transportation

Volume of taxis

Volume of cars

Volume of mototaxis

Number of pot holes

Velocity of cars

Velocity of public transportation

Velocity of taxis

Velocity of mototaxis

Number of traffic violations

Number of lanes

Setting (residential vs commercial)

Setting (avenue vs street)

Presence of crosswalk

Presence of a sidewalk

Density of parked cars

Presence of bus stop

Presence of a park or designated play area

Percentage of sidewalk obstructed

Presence of street light

Presence of police directing traffic

Presence of speed bumps

Road condition (paved vs dirt)

Presence of a gated community

Presence of a stop sign

Drivers using more than the designated number of lanes

Presence of an intact median divider
